# Priming of Soil Carbon Decomposition in Two Inner Mongolia Grassland Soils following Sheep Dung Addition: A Study Using ^13^C Natural Abundance Approach

**DOI:** 10.1371/journal.pone.0078578

**Published:** 2013-11-13

**Authors:** Xiuzhi Ma, Per Ambus, Shiping Wang, Yanfen Wang, Chengjie Wang

**Affiliations:** 1 College of Forestry, Inner Mongolia Agricultural University, Huhhot, China; 2 Department of Chemical and Biochemical Engineering, Technical University of Denmark, Lyngby, Denmark; 3 Laboratory of Alpine Ecology and Biodiversity, Institute of Tibetan Plateau Research, Chinese Academy of Sciences, Beijing, China; 4 Department of Life College, University of Chinese Academy of Sciences, Beijing, China; 5 College of Ecology and Environmental Science, Inner Mongolia Agricultural University, Huhhot, China; Tennessee State University, United States of America

## Abstract

To investigate the effect of sheep dung on soil carbon (C) sequestration, a 152 days incubation experiment was conducted with soils from two different Inner Mongolian grasslands, *i.e.* a *Leymus chinensis* dominated grassland representing the climax community (2.1% organic matter content) and a heavily degraded *Artemisia frigida* dominated community (1.3% organic matter content). Dung was collected from sheep either fed on *L. chinensis* (C_3_ plant with δ^13^C = −26.8‰; dung δ^13^C = −26.2‰) or *Cleistogenes squarrosa* (C_4_ plant with δ^13^C = −14.6‰; dung δ^13^C = −15.7‰). Fresh C_3_ and C_4_ sheep dung was mixed with the two grassland soils and incubated under controlled conditions for analysis of ^13^C-CO_2_ emissions. Soil samples were taken at days 17, 43, 86, 127 and 152 after sheep dung addition to detect the δ^13^C signal in soil and dung components. Analysis revealed that 16.9% and 16.6% of the sheep dung C had decomposed, of which 3.5% and 2.8% was sequestrated in the soils of *L. chinensis* and *A. frigida* grasslands, respectively, while the remaining decomposed sheep dung was emitted as CO_2_. The cumulative amounts of C respired from dung treated soils during 152 days were 7–8 times higher than in the un-amended controls. In both grassland soils, ca. 60% of the evolved CO_2_ originated from the decomposing sheep dung and 40% from the native soil C. Priming effects of soil C decomposition were observed in both soils, *i.e.* 1.4 g and 1.6 g additional soil C kg^−1^ dry soil had been emitted as CO_2_ for the *L. chinensis* and *A. frigida* soils, respectively. Hence, the net C losses from *L. chinensis* and *A. frigida* soils were 0.6 g and 0.9 g C kg^−1^ soil, which was 2.6% and 7.0% of the total C in *L. chinensis* and *A. frigida* grasslands soils, respectively. Our results suggest that grazing of degraded Inner Mongolian pastures may cause a net soil C loss due to the positive priming effect, thereby accelerating soil deterioration.

## Introduction

The availability of soil organic carbon (C) for microbial decomposition is crucial for many processes within the C cycle, and assessment of soil dynamics is of great concern in terms of climate change and soil fertility [1]. Animal dung returned to soil can constitute important source of C, and maintain long-term soil fertility in grassland ecosystems [2]–[4]. However, dung application can also potentially increase soil respiration [5]–[8]. Studies have shown that the addition of easily degradable C to soil may stimulate microbial activity to such an extent that the turnover of soil organic matter (SOM) is accelerated temporarily, an effect that is frequently called the priming effect (PE) [3], [9]–[12]. When a positive PE occurs, the addition of material such as animal slurry to soils may not result in a net C sequestration, but rather a net C loss [9]. The intensity, direction, and extent of PE depends on several parameters, including the amount and quality of added C, soil microbial activity and community structure [13]–[15], soil pH [16] and aggregate size [17].

Distinct signatures in ^13^C content between native soil C and ‘new’ introduced labile C compounds added in the form of animal slurry or manure enables quantification of the interaction in C turnover between different C pools [3], [18]–[20]. By this means, researchers have shown that incorporated slurry-C was lost twice as fast as the native soil C in two soils with different C contents. Slurry incorporation induced a PE, which was most pronounced in the soil with the highest C content [18]. Following the application of slurries with different particle sizes to a grassland soil, significant increases of soil CO_2_ effluxes (by 2–8 times) were observed in all slurry fractions and the highest was found in the smaller slurry particles [3]. In a study with additions of different substrate quality combinations and C-13 characteristics, Kuzyakov & Bol (2004) have identified three distinct C sources for soil CO_2_ emissions and observed that addition of labile C (sugar) lead to changes in SOM [21]. The ^13^C natural abundance trace technique has also been applied in the two-phase model of CO_2_ emission after dairy or pig slurry application, the first phase (0–48 h) dominated by the incorporation of labile slurry C from the liquid phase, while beyond 48 h slurry-derived C was mainly from less mobile particulate C [12], [18], [22]–[23]. However, whereas previous studies mainly focused on the decomposition of cattle dung or slurry [3], [5], [6], [24] or pig slurry [25], less information is available concerning decomposition of sheep faeces C [26].

Inner Mongolia's grasslands in Northern China are representative of large areas of the Eurasian steppe belt [27]. Sheep is the primary livestock in Inner Mongolia's grassland and large amount of sheep dung is applied as fertilizer except for cooking energy [28]. More than 70% of the natural Inner Mongolian grassland area is extensively degenerated as a consequence of increases in livestock numbers and the change of farming systems during the last three decades [28]. Knowledge regarding the importance of sheep dung excretion for soil C cycling in this ecosystem remains sparse. Addition of artificial sheep excreta to Inner Mongolian steppe in autumn did not impact soil microbial biomass C, but microbial activity significantly increased [29]–[30]. None of these studies, however, have achieved detailed information about the fates of sheep dung derived C in the Inner Mongolian grassland soils, and to what extent heavy grazing by sheep, and thus deposition of dung C, will affect the overall soil C balance through increased sequestration or losses due to priming.

We hypothesized that sheep dung additions to Inner Mongolian grassland soils i) cause a positive priming effect on soil C turnover, and ii) lead to differentiated net C loss in soils with contrasting SOM content. The effects of interaction between soil type and sheep dung addition on soil respiration and soil C sequestration were investigated using the ^13^C natural abundance technique through a five-month incubation experiment.

The objectives of the study were (1) to assess the input of sheep dung-derived C to the soil C-pool; (2) to examine the extent of SOM priming due to application of dung C; and (3) to quantify the net C pool changes in soils subject to intensive sheep dung application.

## Materials and Methods

### Ethics Statement

There was no activities involved the endangered or protected species in this study, and Sheep (vertebrates) were involved in this study, so the permission to use sheep in this study was granted from local authority (Dr. Yongfei Bai, Director of Inner Mongolia Grassland Research Station) before we took the soil sample in the field.

### Field site details

Soil incubated in the study was taken from the Inner Mongolia Grassland Ecosystem Research Station, Chinese Ecosystem Research Network (IMGERS, 116°42′E, 43°38′N). This region is part of the temperate semiarid steppe belt of Eurasia. The mean annual temperature is slightly above zero (0.8°C) with a January mean of −21°C (absolute minimum −42°C) and a July mean of 19°C (absolute maximum 39°C). Mean annual precipitation is 330.3 mm but fluctuates greatly among years, and most rainfall events occur in July and August (both means for years 1982–2007; IMGERS weather data). The annual frost-free period generally lasts 90–110 days [31].

### Field sampling, dung collection and preparation

The soil was collected from two grassland sites. One site was characterized by *Leymus chinensis* vegetation (C_3_ plant), which is a dominant species of the climax grassland community in the Inner Mongolian steppe [32]. Under moderate grazing, *L. chinensis* vegetation is replaced by *Cleistogenes squarrosa* (C_4_ plant), which in turn is replaced finally by *Artemisia frigida* (C_3_ plant) vegetation under heavy grazing [33]–[34]. About 87% of plants in this geographical region possess a C_3_ photosynthetic pathway [33], and consequently the ^13^C isotopic signature in soil organic carbon reflects a C_3_ dominated community (Table 1). Further details on the site conditions are given by Chen & Wang [32].

**Table 1:** Physical and chemical characteristics of the two grassland soils used in the incubation study.

Soil	*L. chinensis* soil	*A. frigida* soil
Dominant vegetation	*Leymus chinensis, Stipa grandis*	*Artemisia frigida, Cleistogenes squarrosa*
Soil type	Dark chestnut	Light chestnut
Soil texture	Silty loam	Sandy
Soil organic matter (SOM) (%)	2.1±0.06	1.3±0.05
Soil total N content (g kg^−1^)	1.9±0.1	1.3±0.1
Soil microbial biomass C (mg/kg^−1^)	390±13	251±17
pH (water: soil = 2.5:1)	6.3±0.02	6.6±0.04
δ^13^ value (‰ vs VPDB)	−22.2±0.1	−22.4±0.8

Numbers are mean ± SE of n = 5 replicate bulk soil samples.

Soils were collected from 0–10 cm depth in the two grasslands. Five 10×10 m^2^ plots situated 2 m apart were sampled randomly using a 6 cm diameter auger to achieve ca. 10 kg of soil from each plot. Soil was composited into one bulk sample, vegetation and coarse roots were removed by hand, and the soil was sieved to pass a 2 mm mesh and stored at <5°C under field moist conditions until it was used.

Sheep dung was collected from twenty Mongolian sheep (two-year old), housed in metabolic cages with the approval of the Chinese Experimental Animal Committee of the Chinese Academy of Sciences and the owner of sheep. Dung collection did not influence sheep feeding but limited their activities freely in successive ten days by the metabolic cages. The sheep were divided randomly into two groups (ten for each group), and one group was fed on *L. chinensis*, while another group was fed on *C. squarrosa*. After 5 days trial to allow for equilibration of the ^13^C content in the digestive tract, dung was collected twice per day in plastic bags which were attached to the tails of the sheep. Dung samples were collected during the following five consecutive days. The dung was kept frozen (−20°C) until used. The difference of the C_3_ and C_4_ sheep dung are given in Table 2. There was no significant different of C, N content and C/N ratios for C_3_ and C_4_ sheep dung.

**Table 2:** Characteristics of sheep dung used in the incubation study. Dung was collected from sheep either fed on L. *chinensis* (C_3_ dung) or C. *squarrosa* (C_4_ dung).

Dung type	Dry matter	Organic C	Total N	C/N	Dry matter	Total C	δ^13^ value
	(% w:w)	(% of DM)			(Per 60 g fresh material) a		(‰ vs VPDB)
C_3_ dung	86.1±0.4	43.9±0.3^b^	1.4±0.08	31.3±0.1	51.7±0.4	22.7±0.15	−26.2±0.04
C_4_ dung	85.1±0.6	44.4±0.2	1.29±0.03	34.4±0.1	51.1±0.2	22.7±0.18	−15.7±0.06

a The content based on 60g fresh dung portions as applied in the experiment.

b Numbers are mean ± SE of n = 3 replicates.

### Experimental setup

The incubation experiment was conducted over a 5-month period. Before the start of incubation, soil was mixed thoroughly and the soil moisture was adjusted with demineralized water to 40% of water-holding capacity (WHC). The sheep dung was thawed and homogenized by hand before being mixed into the soil. The incubation experiment included six treatments, i.e. the full combination of the two soils and three applications of sheep dung (C_3_, C_4_ and no addition). Dung was added as 60 g fresh weight portions mixed thoroughly with 1 kg of soil (air-dried equivalent). The soil-dung mixtures were transferred to 2 l Kilner jars in triplicate that were gently tapped on the lab bench to compress the soil. Two sets of jars were prepared, one for gas sampling and one for soil sampling. To minimize water losses from the soils, jars were covered with perforated Parafilm that was only removed 30 min before gas sampling events. The jars were incubated at 20±1°C in a controlled temperature cabinet throughout the 152 days of the experiment. Water content was held constant by regular watering to weight.

### Headspace sampling for analysis of CO_2_ flux and δ^13^ of CO_2_

Samples for CO_2_ efflux and d^13^C isotopic analysis were collected 16 times on days 1, 2, 4, 6, 9, 14, 19, 24, 41, 55, 71, 83, 100, 121, 137 and 152 after sheep dung amendment. The Kilner jars were sealed gas tight by lids equipped with a rubber septum to allow headspace gas to be sampled by syringe and needle. At each gas sampling event, the headspace was sealed for 30 min and four 10-ml headspace samples were collected every 10 min. The sampling involved a three-step procedure. First, the headspace gas was mixed with a 20-ml sampling syringe several times. Second, a 10-ml gas sample was extracted from the headspace and 5 ml used to pressurize an evacuated 2-ml crimp-sealed vial for the ^13^C isotopic analysis of CO_2_. The residual 5 ml gas sample was analyzed immediately for CO_2_ concentration by gas chromatography using a HP 6890 GC equipped with a Chromosorb 101 column (30°C), He carrier gas and Thermal Conductivity Detection. Gas fluxes were calculated from the change in CO_2_ concentration inside the Kilner jars over the 30 min enclosure period. The relationship between CO_2_ concentrations vs. time was significantly linear (*R^2^* = 0.93). Flux rates were thus calculated from the slope of the linear regression lines and expressed as mg C kg^−1^ soil (DM) day^−1^.

The ^13^C of CO_2_ stored in the 2-ml pressurized vials was determined within 1 week. We used a PreCon (Thermo Scientific, Bremen, Germany) trace gas preparation-concentration unit coupled in continuous flow mode to a Delta PLUS isotope ratio mass spectrometer (IRMS, Thermo Scientific). As laboratory standard we used commercial CO_2_ which had been calibrated against certified ^13^CO_2_ standards (Messer Griesheim, Krefeld, Germany). Samples of the certified standards were also included in each batch of analysis. Results relating to ^13^C characteristics are reported as ‰ vs. VPDB [35].

### Soil sampling and analyses

Soil samples were collected from the jars at days 17, 43, 86, 127 and 152 after dung addition using a 2 cm diameter polyethylene pipe (15 cm long). The subsamples were mixed and the visible small sheep dung was sought out. The larger sheep dung particles were carefully removed by tweezers and the smaller fractions of sheep residues in the soil was absorbed by electrostatic effect, which produced by a polyethylene bottle rubbing against a piece of fabric [36].

Soil samples were weighed in Ag-foil capsules, arranged on a microtiter plate, wetted with water to approximately field capacity, and placed in a desiccator containing a beaker with concentrated (12M) HCl. The carbonates are released as CO_2_ by the acid treatment in 6 to 8 h. The soil samples are then dried at 60°C prior to isotope determination [37]. The soil was then finely ground by a ball mill, and a ca. 30 mg subsample was weighed into a tin combustion cup for determination of total carbon and ^13^C:^12^C ratio following flash combustion on an elemental analyzer (EA 1110, CE Instruments, Milan, Italy) coupled in continuous flow mode to the IRMS.

### Calculations

In this study, we assumed that the C_3_ and C_4_ sheep dung materials go through the same transformation and transport processes, so we can differentiate the carbon source both in soil and CO_2_ efflux based on the different isotope value.

We calculated the percentage of dung-derived C in relation to the total soil C according to equation (1):
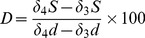
(1)where δ_4_S and δ_3_S are the δ^13^C isotope values of soils amended with C_4_ or C_3_ dung at the time of sampling, and δ_4_d and δ_3_d are the δ ^13^C isotope values of the original C_4_ and C_3_ dung prior to amendment [18], [38]. The difference in δ^13^C between the C_3_ and C_4_ dung in our incubation was 10.5‰ (Table 2).

The fractions of dung-derived C incorporated in the soil at the sampling time in relation to the total dung C applied was calculated with equation (2):
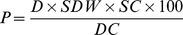
(2)where SDW is soil dry weight, SC is the content of soil organic carbon and DC is the amount of dung C added into soil at the beginning of the incubation.

The difference in δ^13^C values between the respired CO_2_ from the C_4_ and C_3_ dung treatments was used to quantify the proportions of dung versus soil-derived CO_2_-C respired from the soil. The dung-derived CO_2_ was calculated with equation (3):
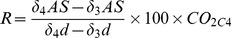
(3)where δ_4_AS-δ_3_AS is the difference in δ^13^C values of CO_2_ emitted from C_4_ dung and C_3_ dung treatments, and CO_2C4_ is the flux of CO_2_ in the C_4_ dung treatment. The approach assumes that any fractionation in ^13^C vs. ^12^C during respiration of sheep dung C and soil C is similar for the C_3_ and C_4_ dung.

### Statistical analyses

Repeated Measures Define Factors of General Linear Model (SPSS 13.0, SPSS Inc. Chicago, Illinois, USA) was used to assess the impacts of treatment, sampling day, soil type and their interactions on the δ^13^C values of soils and CO_2_ emission, the CO_2_ respired efflux, dung-derived carbon, soil-derived carbon and the primed carbon effect. The sampling day was treated as within-subject variables, and soil type and the treatment were used as a between-subject variable. For each observation of CO_2_ emission, cumulative CO_2_ emission, δ^13^C of CO_2_ emission, dung-derived carbon and soil-derived carbon, the significance of differences between treatments was assessed by two-way ANOVA and Least Significance Difference (LSD).

## Results

### Soil carbon derived from sheep dung

Independent of soil type, soil δ^13^C did not differ (p>0.05) between the control soil and the C_3_ dung amended soil throughout the 152 days period (Table 3). In the C_4_ dung soils, δ^13^C significantly exceeded the C_3_ and control treatments from day 127 onwards in the *L. chinensis* soil, and from day 43 onwards in the *A. frigida* soil. The temporal incorporation of C_4_ sheep dung increased soil δ^13^C values by 0.76‰ from day 17 to day 152 in the *L. chinensis* soil, and by 0.46‰ in the *A. frigida* soil (Table 3).

**Table 3:** The dynamics of δ^13^C (Mean ± SE) in control soils and in soil treated with C_3_ and C_4_ dung, and the percent of dung-derived C incorporated in the soil in relation to soil C (D) and applied dung C (P).

Days after addition	Control	δ^13^ C (‰ vs VPDB) C_3_ dung soil C_4_ dung soil		D (% of soil C)	P (% of applied dung C)
*L. chinensis* dominated soil					
17	−22.3±0.1	−22.6±0.1	−22.5±0.02	1.4±0.1	1.3±0.1
43	−22.2±0.1	−22.2±0.1	−22.0±0.1	1.1±0.2	1.2±0.1
86	−22.1±0.01	−22.0±0.04	−22.0±0.1	0.5±0.1	0.5±0.01
127	−22.0±0.1^a^	−22.1±0.1^a^	−21.8±0.1^b^	3.4±0.2	3.2±0.2
152	−22.2±0.1^a^	−22.1±0.3^a^	−21.7±0.1^b^	3.8±0.4	3.5±0.2
*A. frigida* dominated soil					
17	−22.3±0.03	−22.2±0.1	−22.2±0.03	0.03±0.1	0.02±0.00
43	−22.5±0.1	−22.4±0.1	−22.3±0.04	1.0±0.1	0.6±0.01
86	−22.5±1.5^a^	−22.3±0.1^a^	−22.1±0.1^b^	2.1±0.1	1.2±0.04
127	−22.4±0.1^a^	−22.3±0.1^a^	−21.9±0.2^b^	4.0±0.3	2.3±0.1
152	−22.2±0.03^a^	−22.3±0.01^a^	−21.8±0.02^b^	4.9±0.3	2.8±0.2

Data are shown for each sampling during the 152 days incubation.

Different superscript letters represent statistical significance at *P*<0.05 at the same sampling time among treatments.

An increasing amount of dung-derived C appeared in the soil C fractions for both soil types, except for the *L. chinensis* soil at day 86. After 152 days of incubation, about 3.8% and 4.9% of total organic soil C was derived from the applied dung in the *L. chinensis* and *A. frigida* soils, respectively. This was equivalent to 3.5% and 2.8% of the total applied sheep dung C (Table 3).

### Daily and cumulative CO_2_ fluxes

**Figure 1:**
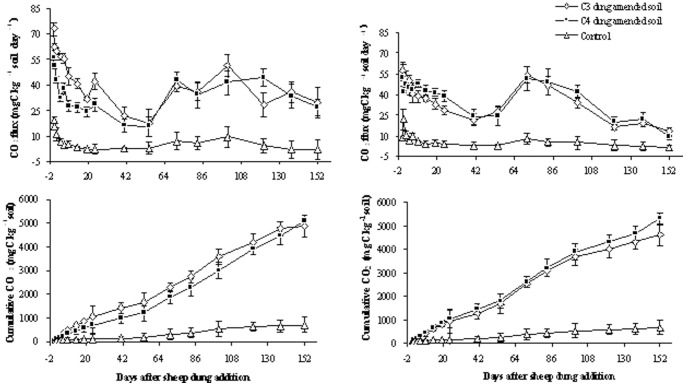
Temporal dynamics of CO_2_ fluxes (mg C kg^–1^ day^–1^) (A and B), and cumulative CO_2_-C loss (mg C kg^–1^ day^–1^) (C and D) during 152 days of incubation of *L. chinensis* and *A. frigida* soils amended with C_3_ and C_4_ dung. Values are the mean (n = 3) ± SE (bars).

The addition of sheep dung to the soil significantly increased CO_2_ flux throughout the experiment in both soils when compared with the control (*P*<0.05; Fig. 1 A, B). There was no difference in CO_2_ emission between the C_3_ dung and C_4_ dung treatments for the two soils, except on days 24 and days 100 in the *L. chinensis* soil, where C_3_ dung amended soil emitted most CO_2_. The two control soils used in the study showed almost uniform CO_2_ emission patterns, maintaining a constant rate (average 3.2 mg C kg^−1^ soil day^−1^) except for the initial increase in CO_2_ flux on days 1–3 which probably resulted from the soil wetting event. A two-phase pattern of soil CO_2_ emission was found in the incubation study for both soils. The first phase was observed during 0–55 days after sheep dung amendment, during which CO_2_ fluxes decreased to 26% and 43% of the initial CO_2_ emission in *L. chinensis* and *A. frigida* soil, respectively. Then a second peak of CO_2_ occurred after 55 days, decreasing again after ca. 100 days (*L. chinensis* soil) and 71 days (*A. frigida* soil) (Fig. 1 A, B). There was no interactive effect between soil type and dung treatment during the incubation.

The cumulative CO_2_ losses from sheep dung amended soils were ca. 7–8 times higher than in the control soils after 152 days (*P*<0.01, Fig. 1 C, D). However, there was no difference in total CO_2_ emission between the C_3_ and C_4_ sheep dung treatments (*P*>0.05, Fig. 1 C, D).

### Isotopic characteristics of emitted CO_2_

**Figure 2:**
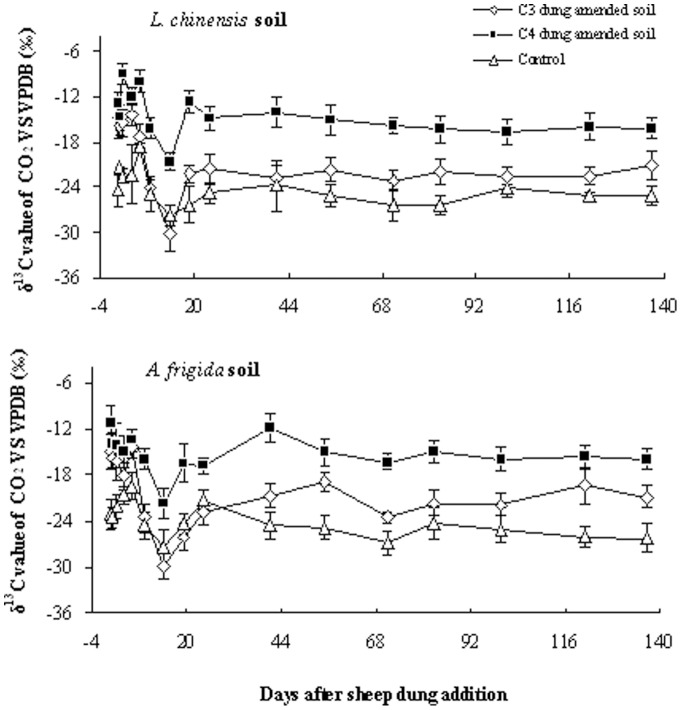
Temporal dynamics of δ^13^C (‰ vs VPDB) signatures in CO_2_ emitted during 152 days following sheep dung addition to *L. chinensis* and *A. frigida* soil (n = 3).

Slightly higher δ^13^C values of CO_2_ were found in all the soils included in the two control soils at the beginning of the experiment. These increased towards a peak value at day 6, and then decreased to a minimum asymptotic value in all treatments at day 14 (Fig. 2). Generally, there was no difference in ^13^C-CO_2_ between the C_3_ dung treatment and control, except at days 55 and 121 for *A. frigida* soil (*P*>0.05). However, the δ^13^C value of CO_2_ from the C_4_ dung treated soils was significantly higher than that in the control soil in most occasions (*P*<0.05). For the C_4_ dung, C_3_ dung, and control treatments, respectively, average δ^13^C of CO_2_ emissions during 152 days incubation were −14.6‰, −20.7‰, and −24.3‰ for *L. chinensis* soil, and −15.2‰, −20.5‰, and −24.0‰ for *A. frigida* soil (Fig. 2).

### CO_2_ emission sources and primed CO_2_ emission in dung amended soil

**Figure 3:**
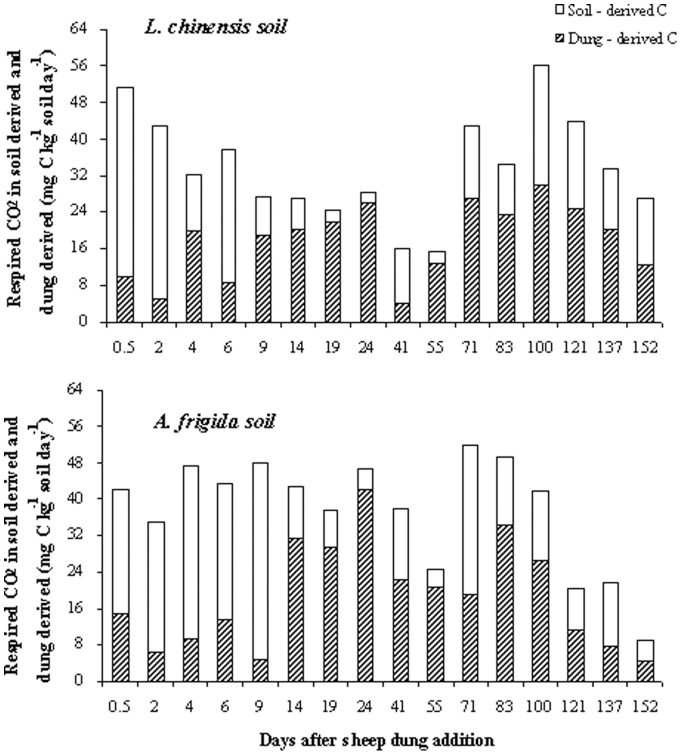
The relative contribution of dung-derived CO_2_-C and soil-derived CO_2_-C during 152 days incubation calculated from the δ^13^ C signature of CO_2_ after sheep dung addition (n = 3).

The simple method of estimating the contribution of dung-derived C in respired CO_2_ is only valid when the respiration rates from the C_3_ and C_4_ dung treated soils are the same[38], as was the case in the current study. Two peaks of dung-derived C were observed in both soils (Fig. 3). As a proportion of the total CO_2_-C respired from the dung treated *L. chinensis* soil, during the first 24 days of the experiment, the dung-derived C increased from 11.5% (day 2) to 90.9% (day 24), and from the dung treated *A. frigida* soil increased from 17.9% (day 2) to 91.0% (day 24) (Fig. 3). The cumulative amount of CO_2_-C respired from the C_4_ dung treatment was 5.11 and 5.33 g C kg^−1^ for *L. chinensis* soil and *A. frigida* soil, respectively (Appendix S1). The amount of dung-derived C recovered in respiration was nearly 1.5 times that from soil-derived C in both soils, *i.e.* 59.7% and 58.9% of the evolved CO_2_ originated from the decomposing dung in the *L. chinensis* soil and *A. frigida* soil, respectively (Fig. 3).

**Figure 4:**
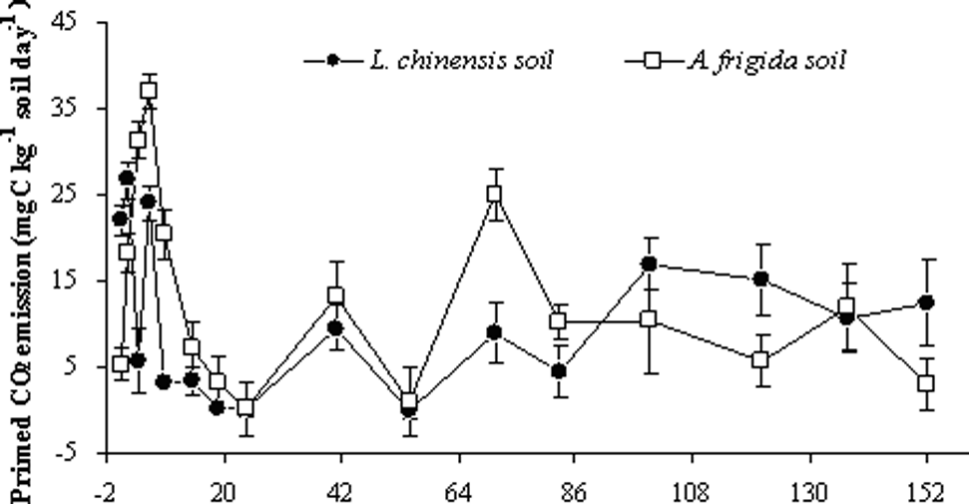
Primed CO_2_ emission (mg C kg^−1^ day^−1^) during 152 days of incubation from sheep dung amended *L. chinensis* and *A. frigida* soils. The numbers indicate soil C derived CO_2_ emitted in excess to the control soil (n = 3).

More CO_2_ emissions in C_4_ dung treated soil than control soil in this study was ascribed to the priming process. The occurrence of positive priming was observed in both soils, with a more pronounced priming effect in *A. frigida* soil than in *L. chinensis* soil (Fig. 4). Thus, compared with the control soils, an additional 1.34 g C (*L. chinensis*) and 1.55 g C (*A. frigida*) was emitted as CO_2_ after sheep dung was applied (Appendix S1 and Table 4).

**Table 4:** Fates of soil and sheep dung carbon after 152

Soil type	Organic C of soil	Fate of sheep dung C		Soil CO_2_ emission (g C kg dry soil^−1^)			Net soil C loss	Total soil C loss
		Recovered in soil	Emitted as CO_2_	Soil derived C	Control	Priming		
*L. chinensis*	21.0±0.06	0.79±0.01	3.05±0.04	2.06±0.01	0.72±0.02	1.34±0.03	0.55±0.01^a^	2.6±0.2^b^
*A. frígida*	13.0±0.05	0.64±0.01	3.14±0.03	2.19±0.01	0.64±0.01	1.55±0.01	0.91±0.02	7.0±0.3

a Net soil C loss was given by the value of sheep dung C sequestrated in the soil during 152 days subtracted from the primed soil CO_2_.

b Soil C loss (%) is the percentage total CO_2_-C loss compared to soil total organic C content.

### Net carbon budget

Over the 152 days incubation period, 3.5% and 2.8% (*i.e.* 0.79 g C and 0.64 g C) of the amended sheep dung was recovered in the *L. chinensis* soil and *A. frigida* soil carbon fractions, respectively (Table 3 and Table 4), and 13.4% and 13.8% of the amended sheep dung was emitted as CO_2_ in the *L. chinensis* soil and *A. frigida* soil carbon fractions, respectively.

A priming effect was found in both soils during the incubation period. Considering the apparent supply of dung C to the soil C component, in comparison with the primed CO_2_ loss from dung treated soil, the net soil C loss was nearly two times higher from the low C *A. frigida* soil (0.91 g C kg^−1^ soil) compared to the high C *L. chinensis* soil (0.55 g C kg^−1^ soil). As a result, 2.6% and 7.0% of soil C was lost due to the application of sheep dung from the *L. chinensis* and *A. frigida* soils, respectively (Table 4).

## Discussion

### Dung-derived C in the soil

The δ^13^C signatures differed by 10.5‰ between the C_4_ and C_3_ sheep dung in our study, which makes it possible to differentiate the soil- and dung-derived C in bulk samples as well as in respired CO_2_ based on the ^13^C natural abundance characteristics. Distinct differences in δ^13^C signatures between C_4_ and C_3_ dung treated (or control) soils emerged 43 days after dung incorporation into *L. chinensis* dominated soil, and 86 days in *A. frigida* dominated soil, suggesting differentiated time lags in the apparent transformation of dung C to the soil C component. However, the limited temporal resolution of soil sampling impeded a detailed identification of the exact temporal dynamics. In contrast, low but significant CO_2_ emissions derived from dung C was observed initially in the incubations, which indicates that dung decomposition commenced immediately, but a transfer to the soil C component was not apparent until after several weeks of incubation.

Bol et al. [38] observed that after a 150 days field experiment, 12.6% of applied cattle dung C was retained in a grassland top soil C component. Another field experiment in a temperate grassland showed that a maximum of 60% of cow dung C was retained in the soil after 56 days, declining to around 20% after 372 days [39]. In our study, only 2.8–3.5% of sheep dung C appeared in the soil C component after 152 days of incubation at 20°C. Probably the relatively low dung water content (*i.e.* 14.5%) in our study caused constrained decomposition compared with other studies (e.g. 84% water content in the study by Bol et al. [38]. Slurry generally decomposes faster than dung due to its liquid nature and missing of various dissolved compounds [40]. The characteristics of the dung, such as its C/N ratio, is also an important factor which affected the decomposition of excreta. The C/N ratios in our experiment (31.3 for C_3_ dung and 34.4 for C_4_ dung) were much higher than the 0.7–10.9 for Bertora et al. [2], and high C/N excreta might be prone to slow mineralization compared to low C/N excreta [2], [41]–[42].

### CO_2_ fluxes

An apparent two-phase pattern of CO_2_ emission was observed in the current study, which was attributed to the two-phase pattern of sheep dung decomposed in both soil.

During the whole period of the incubation (152 days), in both soils, ca 40% of the total CO_2_ was released from dung treated soil itself, while 60% released from the decomposed dung (Fig. 3). The two-stage decomposition patterns have also been observed in other studies on dung decomposition, and it is proposed that in the first stage CO_2_ emission is due to the decomposition of labile C from soil and easily degradable dung fractions, while in the second phase, the decomposers attack more recalcitrant material (3, 18, 37). The first phase of decomposition, as indicated by the increased CO_2_ efflux, lasted for ca. 6 days, which is longer compared with the 24–48 h duration observed in other studies, suggesting that the labile fraction of sheep dung C is more recalcitrant and less available compared to other excreta such as wet cattle dung [24], [29].

### Isotope characteristics of emitted CO_2_

The δ^13^C signal of emitted CO_2_ from both C_4_ incorporated soils were significantly higher than the C_3_ and the control soil in our experiment, which was likely due to the incorporation and the microbial turnover of polymeric biologic cell wall materials from C_4_ dung into C_3_ grassland soils of C_4_ dung C [3]. Similar phenomena have been observed in other studies within the first hours [25]–[26]. For the control soil, in general the δ^13^C of emitted CO_2_ is slightly higher than the δ^13^C of soil undergoing decomposition. For example, Fangueiro et al. [3] reported that the δ^13^C of CO_2_ emitted from untreated soil was on average 5.4‰ higher than the value for the SOM undergoing decomposition. Angers et al. [25] reported 5.0‰ higher δ^13^C of emitted CO_2_ than δ^13^C in the soil itself. At the same time, slightly higher δ^13^C of emitted CO_2_ in the C_4_ dung treated soil (average −14.5‰) than the sheep dung itself (−15.7‰) was found in our incubation, the interaction effect of sheep dung and soil and the isotope fractionation associated with the microbial turnover maybe was the possible reason.

### Soil priming effects after sheep dung addition

A priming effect (PE) is defined as a short-term change in the turnover of soil organic matter caused by the addition of labile organic C to the soil [43]. Here, we determined the priming effect as the excess emissions of soil C derived CO_2_ in dung treated soil compared to control soils. Primed CO_2_ emissions were observed throughout the 152 days incubation in both grassland soils. Specifically, 1.3 g and 1.6 g of excess soil C kg^−1^ was emitted as CO_2_ when dung was added to the *L. chinensis* soil and *A. frigida* soil, respectively, corresponding to 6.2% and 11.9% of total soil C. Such a priming effect is in the upper range of primed C losses of 2.3%–8.9% observed in previous studies [44]–[45].

Priming of soil C decomposition is believed to occur during relatively short-term periods upon addition of labile substrates to soils, and always occurs only in the early stage of substrate addition after which it rapidly ceases [9], [10], [18], [46]. The rapid decrease in the priming effect was likely caused by the depletion of easily decomposable substances added with more complex materials [23]. However, primed soil C decomposition was apparent in our study throughout the entire 152 days period, although with a quantitative variation during the experiment, which may be related to the high C/N ratio and slow decomposition rate characteristics of sheep dung. The priming effect depends not only on the decomposability of the various carbon pools in the environment, but also on the state of the microorganisms [47]–[48], and involves not only one mechanism but rather a succession of processes partly connected with succession of microbial communities and functions [13]. Further research is needed to test the fundamental processes and mechanisms involved in the priming effect of soil organic matter decomposition due to grazing in Inner Mongolian grasslands.

The loss of sheep dung C via CO_2_ was the same in the two grassland soils in our experiment, which contrasts with results by Bol et al. [18] who reported that more slurry-derived C was respired from a C-rich soil compared to a C-poor soil during 0–9 days after slurry incorporation. The authors of that study attributed this to the more pronounced enhancement of basal soil respiration in C-rich soil compared to the C-poor soil. Furthermore, after labile organic C addition, energy limitation in C-poor soil may have ceased, which subsequently facilitated more activation of soil microorganisms, and more enzymes were produced that were capable of SOM degradation [17], [43].

### Conclusion and implications for grassland management

The addition of C_4_ sheep dung to a C_3_ grassland soil enabled us to successfully trace the fate of dung-derived C in the soil and calculate the soil organic C budget for two soils from the Inner Mongolian steppe. Although sheep dung provided an additional organic carbon source for the grassland soils, a large part was emitted as CO_2_ to the atmosphere. After sheep dung addition, a positive priming effect of soil C decomposition was observed in both a high-C *L. chinensis* soil and a low-C *A. frigida* soil. Therefore, the balance of soil organic carbon storage was negative when sheep dung was mixed into the soil. This effect was more pronounced for the degraded community of *A. frigida* soils, from which more soil C was lost compared with the climax community of *L. chinensis* soils. This finding is contrary to the conventional conception of carbon storage in temperate grassland, which predicts that the livestock excreta applied to grassland soils could return essential nutrients for plant growth and increase fertility and SOM contents.

The results suggest that intensive grazing management in the temperate steppe should be avoided. In Inner Mongolian grasslands, the succession from *L. chinensis* dominated communities to *A. frigida* dominated communities will result in decreased plant productivity and soil carbon inputs because of the decreased litter and root nutrient return [34]. Bearing in mind that complex plant-soil interactions which exist under field conditions have not been considered in our study, and assuming that the present conclusion can be extrapolated to field conditions, a further acceleration of the decreasing soil C pool in degraded grasslands may occur. Under actual grazing conditions, sheep dung may be mixed merely within the very top grassland soils, suggesting that the current calculations based on well-mixed soil and dung may overestimate soil C losses. Future work is thus needed to examine whether the priming effect of sheep dung amendments observed in this study can be extended to field conditions.

## Supporting Information

Appendix S1 (DOC)

## References

[1] Pausch J, Kuzyakov Y (2011) Soil organic carbon decomposition from recently added and older sources estimated by δ^13^C values of CO_2_ and organic matter. Soil Biol Biochem 55: 40–47.

[2] Bertora C, Alluvione F, Zavattaro L, Groenigen JWV, Velthof G, et al (2008) Pig slurry treatment modifies slurry composition, N_2_O and CO_2_ emissions after soil incorporation. Soil Biol Biochem 40: 1999–2006.

[3] Fangueiroa D, Chadwick D, Dixon L, Bol R (2007) Quantification of priming and CO_2_ emission sources following the application of different slurry particle size fractions to a grassland soil. Soil Biol Biochem 39: 2608–2620.

[4] Pineiro G, Paruelo JM, Oesterheld M (2006) Potential long-term impacts of livestock introduction on carbon and nitrogen cycling in grasslands of southern South America. Global Change Biol 12: 1267–1284.

[5] Dungait JA, Bol R, Evershed RP (2005) Quantification of dung carbon incorporation in a temperate grassland following spring application using bulk stable isotope determinations. Isot Environ Healt S 41: 3–11. 10.1080/10256010500053516 15823853

[6] Fangueiro D, Chadwick D, Dixon L, Grilo J, Walter N, et al (2010) Short term N_2_O, CH_4_ and CO_2_ production from soil sampled at different depths and amended with a fine sized slurry fraction. Chemosphere 81: 100–108. 20630560 10.1016/j.chemosphere.2010.06.049

[7] Lin XW, Wang SP, Ma XZ, Xu GP, Luo CY, et al (2009) Fluxes of CO_2_, CH_4_, and N_2_O in an alpine meadow affected by yak excreta on the Qinghai-Tibetan plateau during summer grazing periods. Soil Biol Biochem 41: 718–725.

[8] Ma XZ, Wang SP, Wang YF, Jiang GM, Nyren P (2006) Short-term effects of sheep excreta on carbon dioxide, nitrous oxide and methane fluxes in typical grassland of Inner Mongolia. New Zeal J Agr Res 49: 285–297.

[9] Kuzyakov Y, Friedel JK, Stahra K (2000) Review of mechanisms and quantification of priming effects. Soil Biol Biochem 32: 1485–1498.

[10] Luo Y, Durenkamp M, De Nobili M, Lin Q, Brookes PC (2011) Short term soil priming effects and the mineralisation of biochar following its incorporation to soils of different pH. Soil Biol Biochem 43: 2304–2314.

[11] Ohm H, Hame U, Marschner B (2007) Priming effects in soil size fractions of a podzol Bs horizon after addition of fructose and alanine. J Plant Nutr Soil Sc 170: 551–559.

[12] Rochette P, Angers DA, Chantignya MH, Bertranda N, Cote D (2004) Carbon Dioxide and Nitrous Oxide Emissions following Fall and Spring Applications of Pig Slurry to an Agricultural Soil. Soil Sci Sci AM J 68: 1410–1420.

[13] Blagodatskaya E, Kuzyakov Y (2008) Mechanisms of real and apparent priming effects and their dependence on soil microbial biomass and community structure: critical review. Biol Fert Soils 45: 115–131.

[14] Hamer U, Marschner B (2005) Priming effects in different soil types induced by fructose, alanine, oxalic acid and catechol additions. Soil Biol Biochem 37: 445–454.

[15] Shen J, Bartha R (1996) The priming effect of substrate addition in soil-based biodegradation tests. Appl Environ Microb 62: 1428–1430. 10.1128/aem.62.4.1428-1430.1996 PMC167910 8919805

[16] Pineiro G, Paruelo JM, Oesterheld M (2006) Potential long-term impacts of livestock introduction on carbon and nitrogen cycling in grasslands of southern South America. Global Change Biol 12: 1267–1284.

[17] Ohm H, Hame U, Marschner B (2007) Priming effects in soil size fractions of a podzol Bs horizon after addition of fructose and alanine. J Plant Nutr Soil Sc 170: 551–559.

[18] Bol R, Moering J, Kuzyakov Y, Amelung W (2003) Quantification of priming and CO_2_ respiration sources following slurry C incorporation in two grassland soils with different C content. Rapid Commun Mass Sp 17: 2585–2590. 10.1002/rcm.1184 14648893

[19] Blagodatskaya E, Yuyukina T, Blagodatsky S, Kuzyakov Y (2011) Turnover of soil organic matter and of microbial biomass under C_3_–C_4_ vegetation change: Consideration of ^13^C fractionation and preferential substrate utilization. Soil Biol Biochem 43: 159–166.

[20] Kuzyakov Y, Bol R (2004) Using natural ^13^C abundance to differentiate between three CO_2_ sources during incubation of a grassland soil amended with slurry and sugar. J Plant Nutr Soil Sc 167: 669–677.

[21] Kuzyakov Y, Bol R (2006) Sources and mechanisms of priming effect induced in two grassland soils amended with slurry and sugar. Soil Biol Biochem 38: 747–758.

[22] Chantigny MH, Rochette P, Angers DA (2001) Short-term C and N dynamics in a soil amended with pig slurry and barley straw: a field experiment. Can J Soil Sci 81: 131–137.

[23] Glaser B, Bol R, Preedy N, McTiernan K, Clark M, et al (2001) Tracing slurry-derived carbon and nitrogen in a temperate grassland using δ^13^C and δ^15^N natural abundance. J Plant Nutr Soil Sc 164: 467–474.

[24] Lovell RD, Jarvis SC (1996) Effect of cattle dung on soil microbial biomass C and N in a permanent pasture soil. Soil Biol Biochem 28: 291–299.

[25] Angers DA, Rochettea P, Chantignya MH, Lapierre H (2007) Use of ^13^C abundance to study short-term pig slurry decomposition in the field. Soil Biol Biochem 39: 1234–1237.

[26] Kristiansen SM, Brandt M, Hansen EM, Magid J, Christensen BT (2004) ^13^C signature of CO_2_ evolved from incubated maize residues and maize-derived sheep faeces. Soil Biol Biochem 36: 99–105.

[27] Bai YF, Han XG, Wu JG, Chen ZZ, Li LH (2004) Ecosystem stability and compensatory effects in the Inner Mongolia grassland. Nature 43: 181–184. 10.1038/nature02850 15356630

[28] Tong C, Wu J, Yong S, Yan J, Yong W (2004) A landscape scale assessment of steppe degradation in the Xilin River Basin, Inner Mongolia, China. J Arid Environ 59: 133–149.

[29] Liu ZK, Wang SP, Han JG, Wang YF, Chen ZZ (2004) Changes of soil chemical properties in sheep urine patches in Inner Mongolia steppe. Chinese Journal of Applied Ecology 15(12), 2255–2260. 15825437

[30] Ma XZ, Wang SP, Jiang GM, Haneklaus S, Schnug E, et al (2007) Short-term effect of targeted placements of sheep excrement on grassland in Inner Mongolia on soil and plant parameters. Commun Soil Sci Plan 38: 1589–1604.

[31] Chen WW, Wolf B, Zheng XH, Yao ZS, Butterbach-Bahl K, et al (2011) Annual methane uptake by temperate semiarid steppes as regulated by stocking rates, aboveground plant biomass and topsoil air permeability. Global Change Biol 17: 2803–2816.

[32] Chen ZZ, Wang SP (2000) Chen, Z.Z, Wang, S.P., 2000. Plant response to different grazing intensity in Inner Mongolia grasslands. In: Typical Steppe Ecosystem of China, Science Press, Beijing. 13–21.

[33] Chen L, Michalk DL, Millar GD (2002) The ecology and growth patterns of Cleistogenes species in degraded grassland of eastern Inner Mongolia, China. J Appl Ecol 39: 584–594.

[34] Wang SP, Li YH, Wang YF, Han YH (1998) The succession of *Artemisia frigida* rangeland and multivariation analysis under different stocking rates in Inner Mongolia. Acta Agrestia Sinica 6: 299–305.

[35] Formanek P, Ambus P (2004) Assessing the use of δ^13^C natural abundance in separation of root and microbial respiration in a Danish beech (Fagus sylvatica L.) forest. Rapid Commun Mass Sp 18: 897–902. 10.1002/rcm.1424 15095359

[36] Bao SD (2000) Agrochemical Analysis of Soil, Fourth ed. Agriculture Press of China, Beijing.

[37] Harris D, Horwa WR, van Kessel C (2001) Acid fumigation of soils to remove carbonates prior to total organic carbon or carbon-13 isotope analysis. Soil Sci Soc Am J 65: 1853–1856.

[38] Bol R, Kandeler E, Amelung W, Glaser B, Marx M, et al (2003) Short-term effects of dairy slurry amendment on carbon sequestration and enzyme activities in a temperate grassland. Soil Biol Biochem 35: 1411–1421.

[39] Dungait JAJ, Bol R, Bull ID, Evershed RP (2009) Tracking the fate of dung-derived carbohydrates in a temperate grassland soil using compound-specific stable isotope analysis. Org Geochem 40: 1210–1218.

[40] Haynes RJ, Williams PH (1993) Nutrient cycling and soil fertility in the grazed pasture ecosystem. Adv Agron 49: 119–199.

[41] Lupwayi NZ (1999) Leucaena hedgerow intercropping and manure application in the Ethiopian highlands I. Decomposition and nutrient release. Biol Fert Soils 28: 182–195.

[42] Wang SP, Li YH (1997) The influence of different stocking rates and grazing periods on the chemical components in feces of grazing sheep and relationship among the fecal components. Chinese Journal of Animal Nutrition 9: 49–56.

[43] Kuzyakov Y (2002) Review: Factors affecting rhizosphere priming effects. J Plant Nutr Soil Sc 165: 382–396.

[44] Dendooven L, Bonhomme E, Merckx R, Vlassak K (1998) N dynamics and sources of N_2_O production following pig slurry application to a loamy soil. Biol Fert Soils 26: 224–228.

[45] Flessa H, Beese F (2000) Laboratory estimates of trace gas emissions following surface application and injection of cattle slurry. J Environ Qual 29: 262–268.

[46] Kuzyakov Y, Yilmaz G, Stahr K (1999) Decomposition of plant residues of Lolium perenne in soils and induced priming effects under different land use. Agribiological Research 52: 25–34.

[47] Kuzyakov Y, Bol R (2005) Three sources of CO_2_ efflux from soil partitioned by ^13^C natural abundance in an incubation study. Rapid Commun Mass Sp 19: 1417–1423. 10.1002/rcm.1938 15880635

[48] Kuzyakov Y (2010) Priming effects: Interactions between living and dead organic matter. Soil Biol Biochem 42: 1363–1371.

