# Supplementary material for: Priming of Soil Carbon Decomposition in Two Inner Mongolia Grassland Soils following Sheep Dung Addition: A Study Using 13C Natural Abundance Approach
Source: PLoS One. 2013 Nov 13;8(11):e78578. doi: 10.1371/journal.pone.0078578 (PMC3827246; doi:10.1371/journal.pone.0078578)
Supplement: Appendix S1 — (DOC) [file pone.0078578.s001.doc]

**Appendix S** The source and amount of CO_2_ respired from the two soils after sheep dung amended

| Days | Respired CO_2_  (mg C kg^-1^ soil day^-1^)  Control C_3_ dung C_4_ dung | | | | δ^13^ C  C_4_-C_3_  (‰) | Respired CO_2_ in C_4_ dung treatment priming factor | | | |
| --- | --- | --- | --- | --- | --- | --- | --- | --- | --- |
|  |  |  |  |  |  | (mg C kg^-1^ soil day^-1^)  Dung-derived soil-derived primed soil derived / control | | | |
| ***L. chinensis* soil** (%) | | | | | | | | | |
| 1 | 15.59 | 72.89 | 41.45 | / | | / | / | / | / |
| 2 | 19.38 | 56.48 | 51.32 | 2.03 | | 9.92 | 41.40 | 22.02 | 2.14 |
| 3 | 11.22 | 55.73 | 42.88 | 1.21 | | 4.92 | 37.95 | 26.73 | 3.38 |
| 4 | 6.59 | 59.53 | 32.18 | 6.48 | | 19.87 | 12.31 | 5.72 | 1.87 |
| 6 | 4.97 | 62.33 | 37.81 | 2.45 | | 8.81 | 29.00 | 24.03 | 5.83 |
| 9 | 5.34 | 45.04 | 27.53 | 7.23 | | 18.94 | 8.58 | 3.24 | 1.61 |
| 14 | 3.04 | 40.76 | 26.86 | 8.00 | | 20.45 | 6.41 | 3.36 | 2.11 |
| 16 | 2.14 | 31.50 | 24.31 | 9.44 | | 21.86 | 2.45 | 0.31 | 1.15 |
| 24 | 2.31 | 42.22 | 28.46 | 9.55 | | 25.87 | 2.59 | 0.28 | 1.12 |
| 41 | 2.51 | 22.11 | 16.04 | 2.68 | | 4.09 | 11.95 | 9.44 | 4.76 |
| 55 | 2.73 | 17.38 | 15.45 | 8.63 | | 12.70 | 2.75 | 0.02 | 1.01 |
| 71 | 7.09 | 39.49 | 42.88 | 6.58 | | 26.86 | 16.01 | 8.93 | 2.26 |
| 83 | 6.05 | 35.75 | 34.20 | 7.25 | | 23.62 | 10.58 | 4.53 | 1.75 |
| 100 | 9.39 | 51.35 | 56.25 | 5.59 | | 29.96 | 26.29 | 16.90 | 2.80 |
| 121 | 4.36 | 28.27 | 44.05 | 5.88 | | 24.65 | 19.39 | 15.04 | 4.45 |
| 137 | 2.22 | 35.64 | 33.48 | 6.44 | | 20.55 | 12.93 | 10.71 | 5.82 |
| 152 | 2.00 | 29.62 | 26.84 | 4.89 | | 12.51 | 14.34 | 12.34 | 7.16 |
| ∑(g) | 0.717 | 4.876 | 5.111 |  | | **3.055** | **2.055** | 1.337 | 2.86 |
| ***A. frigida* soil** | | | | | | | | | |
| 1 | 8.43 | 57.21 | 46.48 | / | | / | / | / | / |
| 2 | 21.77 | 54.00 | 42.00 | 3.74 | | 14.98 | 27.02 | 5.25 | 1.24 |
| 3 | 10.67 | 51.70 | 35.12 | 1.88 | | 6.29 | 28.83 | 18.15 | 2.70 |
| 4 | 6.40 | 50.11 | 47.10 | 2.07 | | 9.26 | 37.84 | 31.44 | 5.91 |
| 6 | 9.64 | 40.78 | 43.44 | 3.22 | | 13.34 | 30.10 | 20.46 | 3.12 |
| 9 | 6.17 | 38.47 | 47.90 | 1.00 | | 4.58 | 43.32 | 37.15 | 7.02 |
| 14 | 4.18 | 37.04 | 42.78 | 7.69 | | 31.33 | 11.44 | 7.26 | 2.74 |
| 16 | 4.84 | 32.95 | 37.59 | 8.25 | | 29.53 | 8.06 | 3.21 | 1.66 |
| 24 | 4.11 | 22.59 | 46.56 | 9.55 | | 42.35 | 4.21 | 0.10 | 1.03 |
| 41 | 2.48 | 21.61 | 38.00 | 6.17 | | 22.33 | 15.67 | 13.19 | 6.31 |
| 55 | 2.51 | 28.07 | 24.49 | 8.98 | | 20.94 | 3.54 | 1.04 | 1.41 |
| 71 | 7.80 | 54.24 | 51.84 | 3.84 | | 18.96 | 32.88 | 25.08 | 4.22 |
| 83 | 5.06 | 46.58 | 49.32 | 7.24 | | 34.02 | 15.30 | 10.24 | 3.02 |
| 100 | 4.98 | 34.16 | 41.67 | 6.65 | | 26.39 | 15.29 | 10.31 | 3.07 |
| 121 | 3.01 | 16.26 | 20.14 | 5.90 | | 11.32 | 8.81 | 5.80 | 2.93 |
| 137 | 2.04 | 18.62 | 21.84 | 3.78 | | 7.87 | 13.97 | 11.93 | 6.86 |
| 152 | 1.71 | 12.75 | 8.96 | 4.95 | | 4.23 | 4.74 | 3.02 | 2.76 |
| ∑(g) | 0.644 | 4.624 | 5.380 |  | | **3.143** | **2.191** | 1.547 | 3.40 |
